# Analysis of Industrial *Bacillus* Species as Potential Probiotics for Dietary Supplements

**DOI:** 10.3390/microorganisms11020488

**Published:** 2023-02-16

**Authors:** Beata Łubkowska, Joanna Jeżewska-Frąckowiak, Michał Sroczyński, Magdalena Dzitkowska-Zabielska, Aleksandra Bojarczuk, Piotr M. Skowron, Paweł Cięszczyk

**Affiliations:** 1 Faculty of Physical Education, Gdansk University of Physical Education and Sport, K. Gorskiego 1, 80-336 Gdansk, Poland; magdalena.dzitkowska-zabielska@awf.gda.pl (M.D.-Z.); aleksandra.bojarczuk@awf.gda.pl (A.B.); pawel.cieszczyk@awf.gda.pl (P.C.); 2 Department of Molecular Biotechnology, Faculty of Chemistry, University of Gdansk, Wita Stwosza 63, 80-308 Gdansk, Poland; j.jezewska-frackowiak@ug.edu.pl (J.J.-F.); michal.sroczynski@phdstud.ug.edu.pl (M.S.); piotr.skowron@ug.edu.pl (P.M.S.)

**Keywords:** bimodal bacteria, pathogenic bacteria, antagonistic activities, antibiotics susceptibility, bacteriophage, *Bacillus*, probiotics, dietary supplements

## Abstract

So far, *Bacillus* species bacteria are being used as bacteria concentrates, supplementing cleaning preparations in order to reduce odor and expel pathogenic bacteria. Here, we discuss the potential of *Bacillus* species as ‘natural’ probiotics and evaluate their microbiological characteristics. An industrially used microbiological concentrates and their components of mixed *Bacillus* species cultures were tested, which may be a promising bacteria source for food probiotic preparation for supplementary diet. In this study, antagonistic activities and probiotic potential of *Bacillus* species, derived from an industrial microbiological concentrate, were demonstrated. The cell free supernatants (CFS) from *Bacillus licheniformis* mostly inhibited the growth of foodborne pathogenic bacteria, such as *Escherichia coli* O157:H7 ATCC 35150, *Salmonella Enteritidis* KCCM 12021, and *Staphylococcus aureus* KCCM 11335, while some of *Bacillus* strains showed synergistic effect with foodborne pathogenic bacteria. Moreover, *Bacillus* strains identified by the MALDI TOF-MS method were found sensitive to chloramphenicol, kanamycin, and rifampicin. *B. licheniformis* and *B. cereus* displayed the least sensitivity to the other tested antibiotics, such as ampicillin, ampicillin and sulfbactam, streptomycin, and oxacillin and bacitracin. Furthermore, some of the bacterial species detected extended their growth range from the mesophilic to moderately thermophilic range, up to 54 °C. Thus, their potential sensitivity to thermophilic TP-84 bacteriophage, infecting thermophilic *Bacilli*, was tested for the purpose of isolation a new bacterial host for engineered bionanoparticles construction. We reason that the natural environmental microflora of non-pathogenic *Bacillus* species, especially *B. licheniformis*, can become a present probiotic remedy for many contemporary issues related to gastrointestinal tract health, especially for individuals under metabolic strain or for the increasingly growing group of lactose-intolerant people.

## 1. Introduction

A considerable group of bacterial probiotics is based on the *Bacillus* genus, with commonly mentioned *B. subtilis*, *B. coagulans*, *B. pumilus*, *B. licheniformis*, and *B. clausii* [1]. These species are a field of rising scientific interest and they offer a potential for industrial applications, being commonly found in the Asian and West African *Bacillus* fermented foods (BFFs), based on soybeans and locust beans [2]. *Bacillus* species are also promising and particularly important for an fast growing group of lactose-intolerant individuals. The species are typically found in non-dairy fermented products, for example in such traditional fermented foods as Japanese natto (fermented soybeans), Korean kimchi (fermented vegetables, mainly cabbage), and Vietnamese fish sauce as well as in drinks, juices, and on raw and unprocessed fruits and vegetables [3,4]. *Bacillus* species are an alternative to sustain the everyday microbiological balance in human organisms deprived of lactic acid bacteria (LAB) strain sources.

The knowledge of the physiology and biochemistry of bacteria of the genus *Bacillus* as well as the availability of many genetic engineering tools that allow to “manage” their metabolism, facilitates the use of these microorganisms in the food industry [5]. Especially, a special role play α-amylases, which currently account for 25% of all enzyme preparations on the world market. The performed analyzes allow to conclude that the amylases synthesized by *B. licheniformis* and *B. subtilis* show activity in a wide pH range (3.6–10.0) as well as at high temperatures (up to 100 °C) [6].

The *Bacillus* species share sporulation ability, forming one oval endospore per cell. This is a crucial feature for *Bacillus* species to survive environmental stress and harsh conditions of growth, preservation, storage, and distribution. Spore formers show vast tolerance and survivability in extreme temperatures, pH (even bile fluids), salt, dehydration, or poor nutrition [7,8]. Moreover, the *Bacillus* probiotic spore formers are perfect model microorganisms able to survive stabilization methods used in powder product generation such as freeze-drying (lyophilization) or drying, which both involve cell dehydration [9,10]. The present trends in probiotic delivery involve a whole palette of microencapsulation methods, which can significantly increase cell viability during freezing or drying processes [11]. An example of a stabilizing solution for *Bacillus*, but also *Lactobacillus* and *Bifidobacteria*, is vegetable oil (sunflower seed, olive, maize, soya, line seed, sesame, rice) or animal (fish) oil with the addition of polysaccharides, such as maltodextrin or inulin [12].

In this paper, we examinate a microorganisms species devised for industrial and household use, as a potential prototype preparation, possibly subjected certain modifications, for dietary supplementation. The examination includes morphological and quantitative assessment of *Bacillus* species as the second (next to *Lactobacilli*) meaningful group of bacterial probiotics. This potential probiotic product comprises mainly of bimodal bacteria belonging to the *Bacillus* genus, such as *B. subtilis*, *B. atrophaeus*, *B. cereus*, *B. licheniformis*, *B. pumilus*, and *B. amyloliquefaciens*. In this study, we assessed the antagonistic activity of the *Bacilli* strains against foodborne pathogenic bacteria such as *Escherichia coli* O157:H7 ATCC 35150, *Salmonella Enteritidis* KCCM 12021, and *Staphylococcus aureus* KCCM 11335, as well as their probiotic potential. Furthermore, we evaluated the *Bacillus* species for its sensitivity to antibiotics and tested its potential sensitivity to thermophilic TP-84 bacteriophage, infecting thermophilic *Bacilli*, as we are currently carry out a programme for engineering TP-84 bacteriophage bionanoparticles [13,14].

## 2. Materials and Methods

### 2.1. Bacteria, Reagents and Equipment

The microbiological samples were from Piotr Skowron’s private collection. Before analysis, the long-stored samples were thoroughly mixed prior to analysis. The TYM nutrition was prepared from Pepton K [20 g/L] and yeast extract at [4 g/L], which were purchased from BTL Species z o.o. company (Lodz, Poland). At the end, CaCl_2_ [5 mM/L], MgCl_2_ [10 mM/L] from WarChem Sp z o.o. (Warsaw, Poland) and D-fructose [0.5%/L] from BTL Species z o.o. company (Lodz, Poland) were added. For agar TYM plates, 20% agar from BD™ Difco™ company was added. The pH of the microbial mediums was 6.7 (±0.2). All other reagents were purchased from Sigma-Aldrich (St Louis, MO, USA).

### 2.2. Bacterial Isolation and Cultivation

Bacterial biomass from probiotic product was isolated using Sigma 1–14 K microcentrifuge (SciQuip, Wem, UK) and preparative Sigma 3–18 K centrifuge (SciOuip, UK). Bacterial cultivation was conducted in sterilized media, both solid and liquid, in stabilized-temperature incubators (Binder, Tuttlingen, Germany) and Excela E25 incubator-shaker (New-Brunswick Scientific, Edison, NJ, USA). Bacterial biomass was aliquoted into the liquid TYM nutrition in a ratio of 1:9, using serial dilutions from 10^−1^ to 10^−7^. Bacterial cultures on solid agar medium were conducted in the temperature profile ranging for: 37, 40, 45, 48, 51, 54, and 57 °C. Finally, 100 µL of a given dilution were spread on agar surface and incubated for 17 h.

### 2.3. Microscopic and Quantitative Morphological Analysis of Bacteria

Bacterial colonies of *Bacillus* species grown in solid agar culture were counted using a CH-20 colony counter (ChemLand, Starogard, Poland). Bacterial observation was conducted using an MBL 800T light microscope (Olympus, Tokyo, Japan) under 1000× magnification. *Bacillus* strains were identified according to assays previously described: Gram-method, Wirtz staining, and negative Maneval’s method [15,16,17,18].

### 2.4. MALDI-TOF Bacterial Species Identification

Potential probiotic strains’ pure cultures were streaked on TYM agar plates to obtain single colonies and subsequently isolated after 17 h incubation at 37 and 40 °C. The MALDI-TOF method was used for the bacterial species identification [19]. MALDI-TOF mass spectrometry analysis was performed using MALDI Bio-typer (Bruker Daltonics, Billerica, MA, USA) at Medical Laboratories Bruss (Gdynia, Poland). The resulting spectra were compared with intracellular protein profile databases for microbiological species (not shown). Quantification and documentation were conducted using a UV custom Canon EOS documentation system.

### 2.5. Antagonistic Activities

*Escherichia coli* O157:H7 ATCC 35150, *Salmonella Enteritidis* KCCM 12021, and *Staphylococcus aureus* KCCM 11335 were obtained from the University of Gdansk (Gdansk, Poland). All *Bacillus* species strains were grown in TYM medium at 45 °C and pathogenic bacteria were grown in nutrient broth at 37 °C, both 200 rpm. Cell free supernatants (CFS) were collected after the *Bacillus* species strains were cultured in TYM medium for 24 h at 45 °C, then were filtrated used sterile syringe filter (PES 0.45 μm 33 mm). *E. coli* O157:H7 ATCC 35150, *S. Enteritidis* KCCM 12021, and *S. aureus* KCCM 11335 were incubated in the same broth for 24 h and diluted to 0.06 at 600 nm, which is equivalent to the McFarland standard 0,5. Then, 3 mL of each test strain was mixed with 3 mL volume of CFS from the bacterial culture *Bacillus* species and incubated for 37 °C, 200 rpm for 24 h. The growth of foodborne pathogenic bacteria in the presence or absence of CFS was determined at 600 nm.

### 2.6. Antibiotics Susceptibility

Antibiotic susceptibility test of the *Bacillus* species strains was performed using standardized Single-Disc Method, as described previously [20]. All antibiotics, except for bacitracin B10, streptomycin S10, and kanamycin K30, which were purchased from BioMaxima S.A. (Lublin, Polska), used in this study were obtained from Emapol Species z o.o. (Gdansk, Poland). Sources of antibiotics were as follows: kanamycin K30 ug, ampicillin AM10, ampicillin & sulfbactam SAM20, streptomycin S10, rifampicin RA5, oxacillin OX5, bacitracin B10, and chloramphenicol C30. The overnight bacterial cultures were rejuvenated and adjusted to OD = 0.3 (at 600 nm), then were subsequently rubbed onto the surface of TYM agar plates. On 60 mL of the solid medium, 250 µL of the culture of the appropriate probiotic bacteria *Bacillus* species was rubbed. A commercial dispenser was used to apply antibiotic discs. All antibiotic discs were the standard high-concentration discs from 5 to 30 µg/µL. The incubation time was from 20 to 24 h at 37 °C. Zone diameters were measured against a black background illuminated with a high-intensity lamp to provide transmitted light. Inhibition of approximately 80% or more of the growth around a disc was considered to be a zone of inhibition. Some strains exhibited an inner area of light growth immediately around the antibiotic disc with an obvious area of inhibition outside this hazy growth. In these instances, the outer zone of inhibition was measured. The most obvious zone of inhibition was measured in those instances when several concentric zones were observed around an antibiotic disc. Zone diameters of 7 mm did not correspond to any measurable zone of inhibition since the antibiotic discs were between 6 and 7 mm in diameter.

### 2.7. TP-84 Bacteriophage Sensitivity Tests

We tested a potential sensitivity to thermophilic TP-84 bacteriophage, infecting thermophilic *Bacilli*, for the purpose of isolation a new bacterial host for engineered bionanoparticles construction. We tested all 6 species of bacteria that were detected in the tested probiotic by using the MALDI-TOF method. In each case, bacterial lawns were prepared from freshly grown bacterial cultures at OD=0.3 (at 600 nm). 30 µL of the appropriate culture were rubbed onto the surface of solid agar medium and incubated for 1 h. Subsequently, spot tests were performed on these plates with dilutions of bacteriophage TP-84 from 0 to −8. Next, 3 μL of the appropriate dilution was applied to the bacterial lawns. The plates were incubated at the same temperatures as in 2.3, i.e., 37, 40, 45, 48, 51, 54, and 57 °C for 20 h. After this time, observations were made to look for bacteriophage plaques.

### 2.8. Statistical Analysis

All experiments were carried out in triplicate. Data were presented as the mean ± standard deviation (SD) for the indicated number of independently performed experiments. The level *p* < 0.05 was considered statistically significant using one-way analysis of variance (ANOVAs).

## 3. Results

### 3.1. Selection and Quantification of Bacterial Cultures

**Figure 1:**
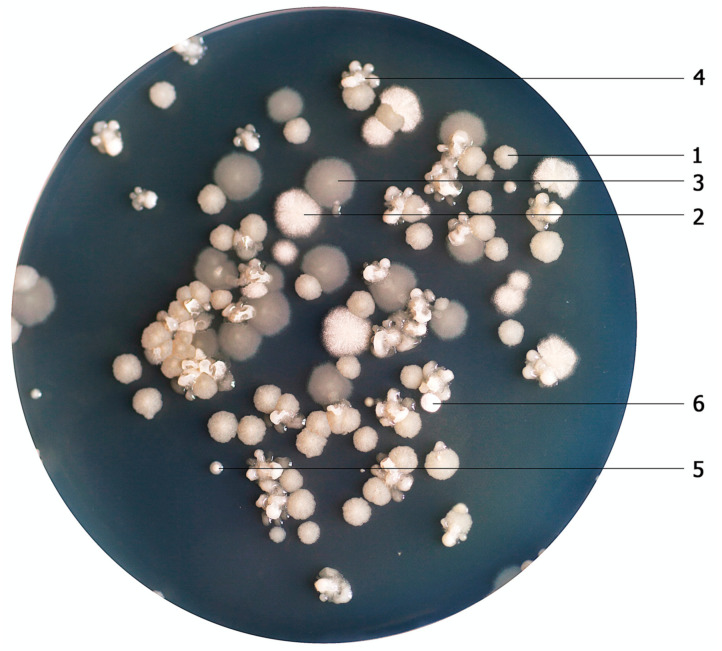
Identification of probiotic bacteria colonies detected in the course of commercial preparation analysis in the growth temperature of 45 °C. Dilution of 10^−7^. Cultures marked: (1) *B. subtilis*, (2) *B. atrophaeus*, (3) *B. cereus*, (4) *B. licheniformis*, (5) *B. pumilus*, (6) *B. amyloliquefaciens*.

**Figure 2:**
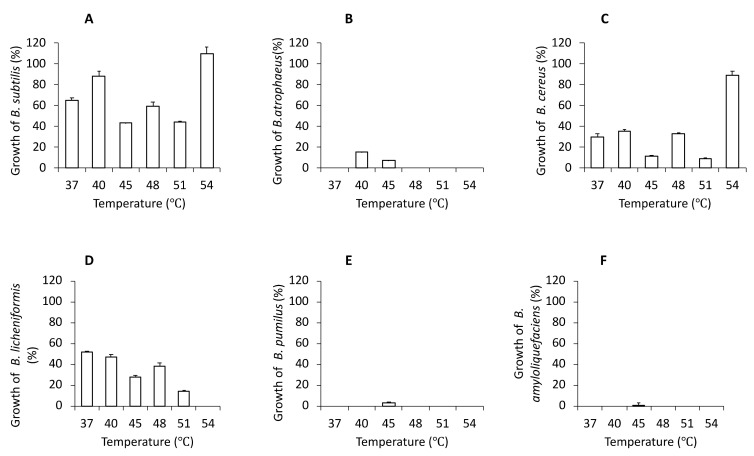
Changes of growth of *Bacillus* strains dependents of temperature range in triplicate. (**A**) *B. subtilis*, (**B**) *B. atrophaeus*, (**C**) *B. cereus*, (**D**) *B. licheniformis*, (**E**) *B. pumilus*, (**F**) *B. amyloliquefaciens*.

We have previously analyzed a few of commercial ‘industrial probiotics-like’ preparations intended for the use as ‘friendly bacteria’ supplements in various cleaning preparations [19]. The use of bacteria cocktails in industrial and household products inevitably leads to their contact with humans. For sheer safety reasons, they are very likely to penetrate the human gastrointestinal tract, their producers had to test them and thus, unintentionally, they provided them as human probiotics. In this study, we have found that some of those species are also common in a numerous other applications, which may be a basis for the development of probiotic food preparation. Six different species of the *Bacillus* group (*B. subtilis*, *B. atrophaeus*, *B. cereus*, *B. licheniformis*, *B. pumilus*, *B. amyloliquefaciens*) were determined, as shown in Figure 1. For the tested product, the temperature 45 °C was used as the most adequate. Environmental *Bacillus* strains were counted quantitatively at a range of temperatures (Supplementary Table S1), while a quantitative analysis of their growth depending on temperature is presented in Figure 2A–F. At a temperature of 40 °C, *Bacillus* strains were less numerous, and fever species were identified. Here, *B. subtilis* dominated, followed by *B. licheniformis* and *B. cereus.* Interestingly, at a temperature of 54 °C, *B. subtilis* and *B. cereus* were the most abundant, which suggests their facultatively thermophilic character despite their fuzzy shape and smaller size of their colonies. None of the *Bacillus* strains grew at 57 °C. Furthermore, the optimal cultivation time for colonies features evaluation, in the temperatures from 37 °C to 57 °C, turned out to be 17 h. A longer incubation time (24 h) resulted in bigger colony size.

Graph A in Figure 2, presents the growth of *B. subtilis* across the entire temperature spectrum from 37 °C to 54 °C, with the highest abundance observed at 54 °C. *B. atrophaeus* grows only at two temperatures: 40 °C and 45 °C (Figure 2B). It is worth noticing that *B. cereus* (Figure 2C) grows to comparable amounts at two temperatures: 37 °C and 48 °C. On the other hand, though *B. cereus* grows most abundantly at 54 °C, its colonies have a ‘blurred’ shape and spread bacterial background. We also determined that *B. licheniformis* grows in the temperature range from 37 °C to 51 °C, as shown in Figure 2D. However, these data could indicate that the number of colonies of *B. licheniformis* decreased when temperature increased. Furthermore, we found out that *B. pumilus* presented in Figure 2E and *B. amyloliquefaciens* presented in Figure 2F only grow as single colonies and only at the temperature of 45 °C. We suggest that perhaps these bacteria may be just a trace contaminant present in the tested *Bacillus* preparations.

### 3.2. Characteristics of Bacterial Cultures, Colonies and Bacterial Cells

**Figure 3:**
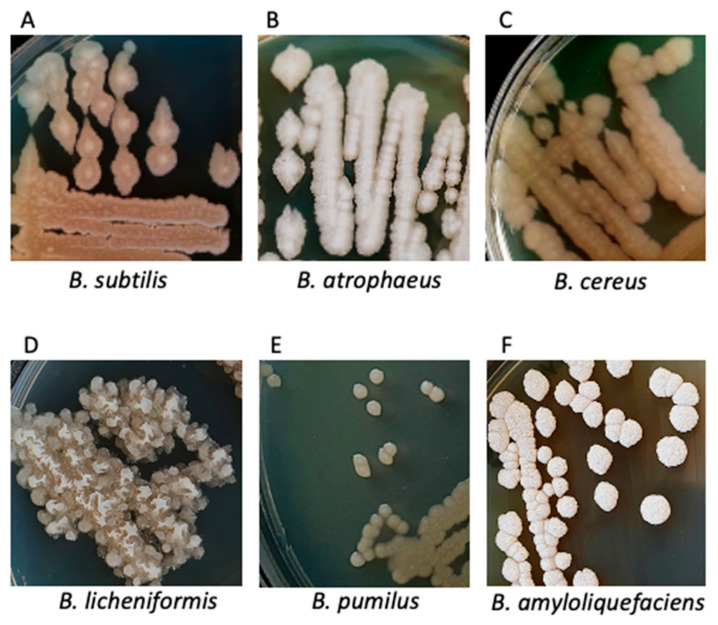
The morphology of bacterial cultures of *Bacillus* species on agar plate.

We also determined the morphology of bacterial cultures, shown in Figure 3A–F. On an agar plate with the well separated colonies of *Bacillus* species, factors such as shape, color, size, and production of extracellular enzymes were examined. As we previously described, the growth of one species of *Bacillus* species in different temperatures was sometimes varied more than between two different species grown in the same conditions. What’s more, as observed under the microscope, this phenomenon occurred regarding the shape and sizes of single cells.

We compared *B. subtilis* colonies (Figure 3A) present to other species of the *Bacillus* group. While *B. subtilis* were round and flat, other *Bacillus* species/strains had more of an irregular shape. Moreover, *B. subtilis* were average in size and had a cream-like color with a lighter dot in the center. In addition, when we incubated at a temperature of 54 °C, the colonies were more transparent and had a white ring (Supplementary Figure S1). This appearance can be explained by the motility characteristics of *B. subtilis*, which seems to increase with higher temperatures, where mucoid or slimy colonies appear [21]. Microscopic study clearly showed that *B. subtilis* are Gram(+)blue-violet rods (Supplementary Figure S2, panel A). Interestingly, heat-resistant endospores of *B. subtilis* were observed in around half of bacteria in the Wirtz staining (Supplementary Figure S2, panel B). The conditions of nutrient limitation drive the population of *B. subtilis* cells to form a mixed population, where half of the population activates the genetic regulator of sporulation and the second half omitted this path, which was demonstrated in previous reports [22]. Furthermore, *B. subtilis* has medium-sized, enveloped mucoid rods, which has been well documented using the Maneval’s tests (Supplementary Figure S2, panel C).

Subsequently, *B. atrophaeus* was examined on nutrition agar (Figure 3B). The results showed large individual colonies (2–5 mm) that had flat and uneven surface covered with a delicate white, non-hemolytic coating with irregular edges. In addition, *B. atrophaeus* were the brightest colored of all tested *Bacillus* species found in this potential probiotic dietary product. Further, our study demonstrated in Gram’s tests that *B. atrophaeus* are Gram(+) purple rods with a pink center (Supplementary Figure S2, panel A). Interestingly, in the Wirtz test, *B. atrophaeus* stained blue but did not show spore-forming. The reason for this is unknown, but we speculated that it is apparently related to the atypical biochemical composition of the spores (Supplementary Figure S2, panel B). Moreover, our Maneval’s study showed that, depending on the growth phase, *B. atrophaeus* formed longer and shorter envelopes with round endospores at the ends (Supplementary Figure S2, panel C).

Previous report by Lim et al. [23] demonstrated that the form of the *B. cereus* colonies varies, depending on the strain. Our study showed that, in the tested *Bacillus* mixtures, *B. cereus* were flat, growing as large single colonies or forming clusters. They were colored gray and/or brown with a lighter center and a wavy edge (Figure 3C). Furthermore, our analyses revealed that there was a wide zone of hemolysis around the colony of *B. cereus*, characteristic of a β-hemolytic bacterium, capable of erythrocytes hemolysis (Supplementary Figure S3). Gram’s staining showed purple Gram(+) rods surrounded by a pink border (Supplementary Figure S2, panel A). While other *Bacillus* rods were short or medium length, *B. cereus* were the longest. Furthermore, after Maneval’s envelopes test, long, thin rods with envelopes were also observed (Supplementary Figure S2, panel C). Wirtz staining showed many endospores of *B. cereus* (Supplementary Figure S2, panel B).

We examined *B. licheniformis* on an nutrition agar (Figure 3D). In this study, numerous clusters of *B. licheniformis* grew, colored white and beige in flower-like in shapes. Despite their medium sizes (3–4 mm), the colonies of *B. licheniformis* built up high to reach to the lid of the plate. Furthermore, cultivated on solid plates with other bacteria (*Bacillus* species mixture diluted 10^−7^), they were attached to other *Bacillus* strains colonies. Further, *B. licheniformis* demonstrated Gram(+) middle size and violet rods morphology (Supplementary Figure S2, panel A). Cultivated in difficult living conditions (temperature above 51 °C) they tended to form spores, while in temperature 37 °C, a vegetative state in Wirtz staining was observed (Supplementary Figure S2, panel B). In addition, in Maneval’s test, *B. licheniformis* exhibited production of envelopes (Supplementary Figure S2, panel C).

Isolated *B. pumilus* colonies were small (1–2 mm), pearly, with concentric growth rings. In addition, *B. pumilus* colonies we distinguishable from outnumbering them other *Bacillus* species only at the temperature of 45 °C upon higher dilution of the bacteria concentrate. After cooling to a temperature of 6 °C, the bacteria turned bright yellow. Hence, we suspect that apparently this treatment induced genes coding for dye production. Interestingly, in the Gram staining analyses, *B. pumilus* appeared in the form of pink rods, and/or pink rods with purple dots inside the bacteria (Supplementary Figure S2, panel A). Later, in the Wirtz test, a large amount of spore-forming were seen (Supplementary Figure S2, panel B). Moreover, in comparison to other strains, oval-shaped colonies of *B. pumilus* were formed with thin envelopes (Supplementary Figure S2, panel C).

In the same way, also on nutrition agar we examined *B. amyloliquefaciens* (Figure 3F). The results showed distinct white-cream colored colonies that built above the surface of the agar plates up to about 1–3 mm. In addition, the colonies had the roughest surfaces and waviest edges of all isolated *Bacillus*. Similarly to *B. pumilus*, *B. amyloliquefaciens* was isolated from the potential probiotic grown at 45 °C as a single colony. Cultivated separately on solid agar medium, it grew in large numbers and released yellow biofilm into the medium (Supplementary Figure S4). Previous reports suggest that *B. amyloliquefaciens* produces γ-polyglutamic acid (γ-PGA) [24]. Our microscope observation indicated that *B. amyloliquefaciens* were long, purple Gram(+) rods with a blue line (Supplementary Figure S2, panel A). Furthermore, the image of green dots indicating spores and blurred shapes was obtained by staining with the Wirtz method (Supplementary Figure S2, panel B). The Maneval test demonstrated that formed clusters, with the envelopes fused together to form a white, irregularly shaped surface (Supplementary Figure S2, panel C).

### 3.3. MALDI-TOF Mass Spectrometry Analysis of Bacteria 

In this study, the microbiological identity of bacterial species/strains present was investigated by microbial protein spectrum (proteome) mass spectroscopy analysis in MALDI-TOF assays as described [25,26]. These data allowed the distinguishing of closely related *Bacillus* species. *B. subtilis*, *B. atrophaeus*, *B. cereus*, *B. licheniformis*, *B. pumilus*, *B. amyloliquefaciens* were determined in the samples of the analyzed product. Reliable identification to the genus and species level using the Bruker MALDI Biotyper mass spectrometer was confirmed. These results indicated, that the value of the strain identification index was respectively: for *B. subtilis*—2.27, *B. atrophaeus*—1.89, *B. cereus*—1.96, *B. licheniformis*—2.22, *B. pumilus—*2.10, *B. amyloliquefaciens*—1.96. Therefore, due to the fact that for three species—*B. atrophaeus*, *B. cereus, and B. amyloliquefaciens*—the confidence factor was slightly below the value of 2.0, the presence of these species in the tested product, although highly probably, leaves a margin of lower confidence.

### 3.4. Antagonistic Effect of Foodborne Pathogenic Bacteria in the Presence of CFS from Bacillus Strains

**Figure 4:**
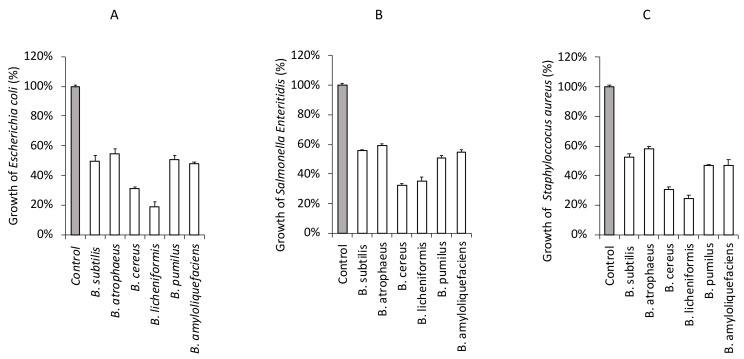
Inhibition of foodborne pathogenic bacterial growth in the presence of CFS from *Bacillus* strains isolated from potential probiotic source. *Escherichia coli* O157:H7 ATCC 35150 (**A**), *Salmonella Enteritidis* KCCM 12021 (**B**), or *Staphylococcus aureus* KCCM 11335 (**C**) were incubated in the presence of CFS from the *Bacillus* strains (*B. subtilis*, *B. atrophaeus*, *B. cereus*, *B. licheniformis*, *B. pumilus*, *B. amyloliquefaciens*) at 37 °C for 24 h. The bacterial growth was determined at OD_600_. The growth rate of foodborne pathogenic bacteria without CFS was assigned to 100% (Control).

As a result of this study, antagonistic activity of CFS from the *Bacillus* strains against foodborne pathogenic bacteria was determined as shown in Figure 4A–C and Supplementary Table S2. All the CFS partially inhibited the growth of *E. coli* O157:H7 ATCC 35150 (Figure 4A), *Salmonella Enteritidis* KCCM 12021 (Figure 4B), and *S. aureus* KCCM 11335 (Figure 4C).

Isolated *B. subtilis* showed antagonistic activity against in about 50% of both gram-positive (*S. aureus*) and gram-negative bacteria (*E. coli*, *S. enteritidis*). This study also revealed a weak antagonistic activity of *B. atrophaeus*. It inhibited the activity of food-borne pathogenic bacteria only in 40%, which indicates that this bacterium is of low functionality as a probiotic bacterium. *B. cereus* exhibits a wide range of zone of inhibition and was bacteriostatic for three of the tested pathogens in 70%. The above result indicates that the isolate might produce some substances that have an inhibitory effect on a wide range of bacteria. The results revealed that *B. licheniformis* exhibited various degrees of inhibitory activities against foodborne pathogenic bacteria, which indicates a broad spectrum of inhibition patterns of this organism. Maximum inhibitory activity was observed against *E. coli* (85%), followed by *S. aureus* (77%) and *S. enteritidis* (68%). *B. pumilus* showed antagonistic activity against in about 50% of foodborne pathogenic bacteria, similarly to *S. subtilis*. On the other hand, *B. amyoliquefaciens* showed antagonistic activity against *E. coli* and *S. aureus* in over 50%, like previous reports indicated [27]. A minor inhibitory effect for *S. enteritidis* (45%) was observed.

To further confirm these results, an agar well diffusion assay was performed using the neutralized CFS (adjusted to pH 3.5 by adding 1 M HCl). Since *Bacillus* produce several antimicrobial compounds including organic acids, bacteriocins, enzymes, and antibiotics, we tested whatever neutralized CFS would be able to inhibit the growth of pathogenic bacteria. However, these results suggest that the neutralized CFS were unable to inhibit the growth of *E. coli* O157:H7 ATCC 35150, *Salmonella Enteritidis* KCCM 12021, and *S. aureus* KCCM 11335 (data not shown). These data could indicate that the inhibition of foodborne pathogenic bacterial growth was due to organic acids or non-bacteriocin compounds from the *Bacillus* strains.

### 3.5. Antibiotic Resistance

**Figure 5:**
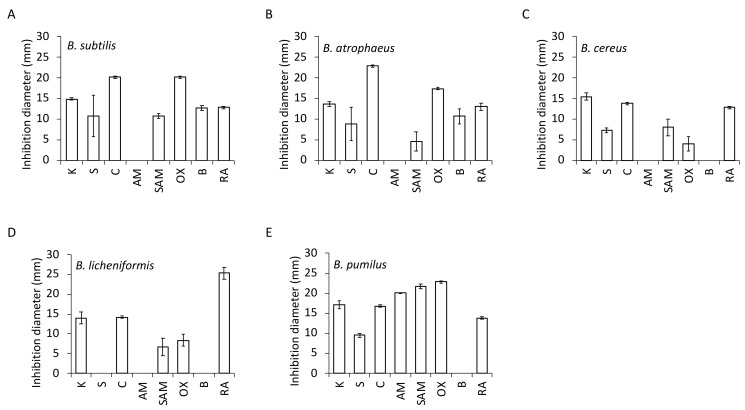
Antibiotic resistance and susceptibility of the *Bacillus* derived from a potential probiotic source. Data for triplicate.

In this study, we determined whether organisms are resistant (R) or susceptible (S) to the antibiotics, as shown in Table 1. The data for triplicate incubation plates with antibiotics are presented on diagrams (Figure 5A–E). In addition, the zones of growth inhibition of *Bacillus* are presented in Supplementary Figure S5A–E and using the interpretative zones diameters given in Supplementary Table S3. All the isolated *Bacillus* species revealed resistance to ampicillin, with one exception—*B. pumilus.*

As displayed in Figure 5A and Supplementary Table S3, *B. subtilis* showed sensitivity to kanamycin, chloramphenicol, oxacillin, bacitracin, rifampicin, and complex of ampicillin and sulfbactam. In addition, for chloramphenicol and oxacillin, we observed particularly large inhibition zones. Another antibiotic, streptomycin-showed ambiguous outcomes because it appeared in a few colonies growing next to the disc of streptomycin, which possibly comprise spontaneous R mutants.

*B. atrophaeus* (Figure 5B, Supplementary Table S3) showed sensitivity to chloramphenicol with the biggest zone of growth inhibition; followed by oxacillin, kanamycin, and rifampicin. On the contrary, bacitracin, streptomycin, and the complex of ampicillin and sulfbactam revealed its resistance or regrowth. The results indicated the appearance of a few colonies of resistant *B. atrophaeus*, or its regrowth, following the decomposition of the antimicrobial agent. 

Furthermore, the study showed that chloramphenicol and rifampicin caused very clear zones of growth inhibition of *B. cereus* (Figure 5C, Supplementary Table S3), while for complex of ampicillin and sulfbactam, streptomycin, and oxacillin, clear zones of inhibition were not recorded. Similar ambiguous results were obtained with kanamycin which, despite causing the largest inhibition zone, left it with small amount of bacteria. 

*B. licheniformis* (Figure 5D, Supplementary Table S3) exhibited sensitivity to chloramphenicol only. Although big zones of growth inhibition of *B. licheniformis* to rifampicin and kanamycin were observed, again regrowth of a few single colonies was evident. This was similar for oxacillin and complex ampicillin and sulfbactam, where the zones of inhibition were very small. *B. licheniformis* was only one bacterium isolated from *Bacillus* species, which proved resistant to three antibiotics: bacitracin, streptomycin and ampicillin. Moreover, the additional brightening zones around the antibiotic discs displayed a pattern very characteristic of *B. licheniformis*, resembling sunflowers.

*B. pumilus* (Figure 5E, Supplementary Table S3) showed sensitivity to all antibiotics, except bacitracin, similarly to *B. cereus* and *B. licheniformis*. *B. pumilus* as the only one from isolated *Bacillus* species gave clear results, as very big zones of growth inhibition without regrowth were observed. Moreover, additional very wide brightening zones around the antibiotic discs, especially for ampicillin and sulfbactam, oxacillin, kanamycin, and ampicillin, were observed.

No zones of inhibited growth for *B. amyloliquefaciens* have been determined due to the very low content of this bacterium in the potential *Bacillus* probiotic source.

### 3.6. TP-84 Bacteriophage Sensitivity Tests

We evaluated the *Bacillus* species identified by MALDI-TOF for potential sensitivity to thermophilic TP-84 bacteriophage, infecting thermophilic *Bacilli*, as we are currently carry out a programme for engineering TP-84 bacteriophage bionanoparticles [13,14]. At 54 °C, only two bacterial species (*B. subtilis*, *B. cereus*) multiplied, while at 51 °C, three of the bacteria detected (*B. subtilis*, *B. cereus*, *B. licheniformis*) multiplied, as shown in Figure 1. Thus they were subjected to TP-84 infections. However, all of them exhibited resistance to TP-84 bacteriophage. 

## 4. Discussion

Commercially available probiotics originate from the gastrointestinal tract of humans and animals as well as from dairy fermented foods. Probiotic *Bacillus* strains, when applied in the form of health foods and dietary supplements or functional feeds and feed supplements, have numerous documented beneficial effects on humans and animals [1]. Although a number of *Bacillus* are involved in the process of fermentation, only *B. subtilis* has been extensively studied as a probiotic agent for purposes such as adhesive characteristics to human intestinal epithelial cells, as well as antioxidant and anti-microbial activities [28]. Thus, in this study, we characterized the antagonistic activities and probiotic potential of *Bacillus* species isolated from a commercial concentrate, and showed their sensitivity/resistance to antibiotics. We confirmed their probiotic usage potential-isolated *B. subtilis* showed antagonistic activity against both gram-positive (*S. aureus*) and gram-negative bacteria (*E. coli*, *S. enteritidis*) (Figure 4). Further analysis provided that *B. licheniformis* exhibited the best degrees of inhibitory activities against the foodborne pathogenic bacteria, with a maximum inhibition degree for *E. coli* (Figure 4).

Each potential probiotic strain has its own specific properties regarding antibiotic resistance. The knowledge of susceptibility and resistance of *Bacillus* strains to antibiotics is an important prerequisite for probiotics formulations designers. Although antibiotic resistance of *Bacillus* strains might be considered a major concern for probiotic application, the determination of antibiotic resistance among *Bacillus* strains is confounded by testing methods [29]. Our study showed that most of the *Bacillus* strains tested were sensitive to chloramphenicol and rifampicin (Table 1, Figure 5 and Supplementary Figure S5). Furthermore, isolated *Bacillus* derived from probiotic were resistant to ampicillin, except one—*B. pumilus*. Still, intrinsic resistance to antibiotics is not considered as a risk to animal and human health [30]. In some cases, such ‘human neutral’ antibiotic resistance may be beneficial during prolonged antibiotic therapies, where existing human microbiome is damaged. In such a case, *Bacillus* species probiotics may temporarily fill in this niche.

*Bacillus* biofilm formation supports the host organism against the gastrointestinal tract (GIT), urogenital tract (UGT), and urinary tract (UT) infections, while modulating immune system activity [2]. Our further analyses revealed that *B. amyloliquefaciens* also form biofilms (Supplementary Figure S4). The balancing effect and favorable colonization by *Bacillus* probiotics are sustained, even if an administered preparation contains spores [31] or if the sporulation occurs in upper parts of the GIT in the stomach or due to bile activity. The *Bacillus* species probiotic biofilm introduces biochemical effects such as antimicrobial and enzymatic activity, thus contributing to protection from dysbiosis as well as GIT and other infections. Moreover, in vitro models of the human stomach and human studies with probiotic *Bacillus* species reveal the mechanisms of its life cycle and sporulation [1,32]. Our microscope observation indicated the present of endospores for all isolated *Bacillus* species (Supplementary Figure S2).

Despite identification of six *Bacillus* species on an agar medium plates (Figure 3) has been demonstrated, spectroscopic analysis identified with high degree of confidence only three out of six initially detected *Bacillus*. Previous reports confirmed that the detected *B. subtilis*, *B. licheniformis,* and *B. pumilus* play the most important role in probiotic bacteria preparations. These bacteria are currently used in the production of food for both humans and animals [33,34,35]. Previous reports demonstrated that one of example of potentially pathogenic *Bacillus* species are *B. cereus and B. anthracis*, as some of its strains produce toxins with different levels of toxicity, posing a human or animal-health risk [28,36]. Thus these bacteria should not be included in the development of bimodal probiotic from *Bacillus* species. However, in the analyzed conglomerate only a small number of *B. cereus* were recorded as growing on a solid medium at temperatures of 40 °C and 45 °C (Figure 2B), and thus they may be an unintended contamination during large scale cultivation of mixed *Bacillus* species population. In addition, *B. cereus* colonies are typically larger, thus this species might actually be a closely related *B. cereus*-like species. Furthermore, it the MALDI-TOF factor of 1.99 is low-to-medium as shown, this isolate may represent a variant of a ‘human-friendly’ species *B. atrophaeus*, containing some more common proteins with *B. anthracis*. Within such a closely related ‘*Bacillus* group’, horizontal gene transfer undoubtedly is a common phenomenon. The above analysis points to the possibility of exploring *Bacillus* preparations used for other than human use purposes for valuable species, which eventually could be used for new formulations as human probiotics upon further extensive testing.

Despite the beneficial activity of *Bacillus* strains belonging to the safety group 1, a number of strains can pose a substantial health risk, carrying genes for various toxins or antibiotic resistance [37]. Furthermore, a promising future application of the probiotic *Bacillus* species might be microflora biocontrol in the human body and the closest human environment. Although the environmental probiotic *Bacillus* species display the potential to support human microflora, the controversies regarding the safety of certain strains is a key factor in their still-limited application [37].

## Figures and Tables

**Table 1:** Antibiotic resistance of the *Bacillus* species derived from a potential probiotic source.

Bacillus Species	Antibiotics							
	K	S	C	AM	SAM	OX	B	RA
*B. subtilis*	S	ND	S	R	S	S	S	S
*B. atrophaeus*	S *	ND *	S *	R *	ND *	S *	ND *	S *
*B. cereus*	ND	ND	S	R	ND	ND	R	S
*B. licheniformis*	ND *	R *	S *	R *	ND *	ND *	R *	ND *
*B. pumilus*	S *	S *	S *	S *	S *	S *	R *	S *
*B. amyloliquefaciens*	ND	ND	ND	ND	ND	ND	ND	ND

Results are shown antibiotics susceptibility. R—means resistant to the antibiotics, no antimicrobial effect; S—sensitivity to the antibiotics, true zone of growth inhibition; ND— ambiguous outcome or potential resistance or regrowth; an asterisk (*) indicates that additional clear zones were visible. Antibiotics discs were as follows: K—kanamycin (30 μg), AM—ampicillin (10 μg), SAM—ampicillin & sulfbactam (20 μg), S—streptomycin (10 μg), RA—rifampicin (5 μg), OX—oxacillin (5 μg), B—bacitracin (10 μg), and C—chloramphenicol (30 μg).

## Data Availability

The authors confirm that the data supporting the findings of this study are available within the article and its supplementary materials.

## Supplementary Materials

The following supporting information can be downloaded at: https://www.mdpi.com/2076-2607/11/2/488/s1, Figure S1: Growth of *B. subtilis* on agar plate in 54 °C; Figure S2: Microscopic analyses of *Bacillus* species cells by Gram staining (panel A), Wirtz staining (panel B) and Maneval tests (panel C); Figure S3: The colony of *B. cereus* with a wide zone of hemolysis after incubated in temperature 45 °C; Figure S4: Colonies of *B. amyloliquefaciens* with a yellow biofilm secreted into the agar medium; Figure S5: Antibiotic sensitivity test of isolated probiotic *Bacillus* derived from the probiotic preparation; Table S1: Quantification of bacterial species in the temperature range tested; Table S2: The antagonistic activity of CFS from the *Bacillus* strains against foodborne pathogenic bacteria; Table S3: Zone of inhibition growth of the *Bacillus sp.* derived from probiotic.
