# Correction: Łubkowska et al. Analysis of Industrial *Bacillus* Species as Potential Probiotics for Dietary Supplements. *Microorganisms* 2023, *11*, 488

**DOI:** 10.3390/microorganisms11071716

**Published:** 2023-06-30

**Authors:** Beata Łubkowska, Joanna Jeżewska-Frąckowiak, Michał Sroczyński, Magdalena Dzitkowska-Zabielska, Aleksandra Bojarczuk, Piotr M. Skowron, Paweł Cięszczyk

**Affiliations:** 1 Faculty of Physical Education, Gdansk University of Physical Education and Sport, K. Gorskiego 1, 80-336 Gdansk, Poland; magdalena.dzitkowska-zabielska@awf.gda.pl (M.D.-Z.); aleksandra.bojarczuk@awf.gda.pl (A.B.);; 2 Department of Molecular Biotechnology, Faculty of Chemistry, University of Gdansk, Wita Stwosza 63, 80-308 Gdansk, Poland; j.jezewska-frackowiak@ug.edu.pl (J.J.-F.); michal.sroczynski@phdstud.ug.edu.pl (M.S.); piotr.skowron@ug.edu.pl (P.M.S.)

## Text Correction

There was an error in the original publication [1]. There was a mistake in our analyzed sample sourse. Mistakenly assigned sample source. The sample originated from a private collection, not the company specified in the uncorrected publication.

A correction has been made to *Abstract, lines 4–5*. The correct text appears below.

“An industrially used microbiological concentrates and their components of mixed *Bacillus* species cultures were tested.”

A correction has been made to *1. Introduction, paragraph 4, lines 1–3*. The correct text appears below.

“In this paper, we examinate a microorganisms species devised for industrial and household use, as a potential prototype preparation, possibly subjected certain modifications, for dietary supplementation.”

A correction has been made to *2.1. Bacteria, Reagents and Equipment*. The correct text appears below. 

*2.1. Bacteria, Reagents and Equipment*

The microbiological samples were from Piotr Skowron’s private collection. Before analysis, the long-stored samples were thoroughly mixed prior to analysis. The TYM nutrition was prepared from Pepton K [20 g/L] and yeast extract at [4 g/L], which were purchased from BTL Species z o.o. company (Lodz, Poland). At the end, CaCl_2_ [5 mM/L], MgCl_2_ [10 mM/L] from WarChem sp z o.o. (Warsaw, Poland) and D-fructose [0.5%/L] from BTL Species z o.o. company (Lodz, Poland) were added. For agar TYM plates, 20% agar from BD™ Difco™ company was added. The pH of the microbial mediums was 6.7 (±0.2). All other reagents were purchased from Sigma-Aldrich (St Louis, MO, USA).

A correction has been made to *3.1. Selection and Quantification of Bacterial Cultures, paragraph 1*. The correct text appears below.

“We have previously analyzed a few of commercial ‘industrial probiotics-like’ preparations intended for the use as ‘friendly bacteria’ supplements in various cleaning preparations [19]. The use of bacteria cocktails in industrial and household products inevitably leads to their contact with humans. For sheer safety reasons, they are very likely to penetrate the human gastrointestinal tract, their producers had to test them and thus, unintentionally, they provided them as human probiotics. In this study, we have found that some of those species are also common in a numerous other applications, which may be a basis for the development of probiotic food preparation. Six different species of the *Bacillus* group (*B. subtilis*, *B. atrophaeus*, *B. cereus*, *B. licheniformis*, *B. pumilus*, *B. amyloliquefaciens*) were determined, as shown in Figure 1. For the tested product, the temperature 45 °C was used as the most adequate. Environmental *Bacillus* strains were counted quantitatively at a range of temperatures (Supplementary Table S1), while a quantitative analysis of their growth depending on temperature is presented in Figure 2A–F. At a temperature of 40 °C, *Bacillus* strains were less numerous, and fever species were identified. Here, *B. subtilis* dominated, followed by *B. licheniformis* and *B. cereus*. Interestingly, at a temperature of 54 °C, *B. subtilis* and *B. cereus* were the most abundant, which suggests their facultatively thermophilic character despite their fuzzy shape and smaller size of their colonies. None of the *Bacillus* strains grew at 57 °C. Furthermore, the optimal cultivation time for colonies features evaluation, in the temperatures from 37 °C to 57 °C, turned out to be 17 h. A longer incubation time (24 h) resulted in bigger colony size.”

A correction has been made to *3.1. Selection and Quantification of Bacterial Cultures, paragraph 2, lines 9–12.* The correct text appears below.

“Furthermore, we found out that *B. pumilus* presented in Figure 2E and *B. amyloliquefaciens* presented in Figure 2F only grow as single colonies and only at the temperature of 45 °C. We suggest that perhaps these bacteria may be just a trace contaminant present in the tested *Bacillus* preparations.”

A correction has been made to *3.2. Characteristics of Bacterial Cultures, Colonies and Bacterial Cells, paragraph 2, lines 1–2*. The correct text appears below.

“We compared *B. subtilis* colonies (Figure 3A) present to other species of the *Bacillus* group.”

A correction has been made to *3.2. Characteristics of Bacterial Cultures, Colonies and Bacterial Cells, paragraph 4, lines 2–3*. The correct text appears below.

“Our study showed that, in the tested *Bacillus* mixtures, *B. cereus* were flat, growing as large single colonies or forming clusters.”

A correction has been made to *3.2. Characteristics of Bacterial Cultures, Colonies and Bacterial Cells, paragraph 5, line 5*. The correct text appears below.

“(*Bacillus* species mixture diluted 10^−7^).”

A correction has been made to *3.2. Characteristics of Bacterial Cultures, Colonies and Bacterial Cells, paragraph 6, lines 2–3*. The correct text appears below.

“In addition, *B. pumilus* colonies we distinguishable from outnumbering them other *Bacillus* species only at the temperature of 45 °C upon higher dilution of the bacteria concentrate.”

A correction has been made to *3.3. MALDI-TOF Mass Spectrometry Analysis of Bacteria*. The correct text appears below.

“In this study, the microbiological identity of bacterial species/strains present was investigated by microbial protein spectrum (proteome) mass spectroscopy analysis in MALDI-TOF assays as described [25,26]. These data allowed the distinguishing of closely related *Bacillus* species. *B. subtilis*, *B. atrophaeus*, *B. cereus*, *B. licheniformis*, *B. pumilus*, *B. amyloliquefaciens* were determined in the samples of the analyzed product. Reliable identification to the genus and species level using the Bruker MALDI Biotyper mass spectrometer was confirmed. These results indicated, that the value of the strain identification index was respectively: for *B. subtilis*—2.27, *B. atrophaeus*—1.89, *B. cereus*—1.96, *B. licheniformis*—2.22, *B. pumilus*—2.10, *B. amyloliquefaciens*—1.96. Therefore, due to the fact that for three species—*B. atrophaeus*, *B. cereus*, and *B. amyloliquefaciens*—the confidence factor was slightly below the value of 2.0, the presence of these species in the tested product, although highly probably, leaves a margin of lower confidence.”

A correction has been made to *3.5. Antibiotic Resistance, paragraph 7*. The correct text appears below.

“No zones of inhibited growth for *B. amyloliquefaciens* have been determined due to the very low content of this bacterium in the potential *Bacillus* probiotic source.”

A correction has been made to *4. Discussion, paragraph 1, lines 7–10*. The correct text appears below. 

“Thus, in this study, we characterized the antagonistic activities and probiotic potential of *Bacillus* species isolated from a commercial concentrate, and showed their sensitivity/resistance to antibiotics.”

A correction has been made to *4. Discussion, paragraph 4, lines 8–21*. The correct text appears below. 

“Thus these bacteria should not be included in the development of bimodal probiotic from *Bacillus* species. However, in the analyzed conglomerate only a small number of *B. cereus* were recorded as growing on a solid medium at temperatures of 40 °C and 45 °C (Figure 2B), and thus they may be an unintended contamination during large scale cultivation of mixed *Bacillus* species population. In addition, B. cereus colonies are typically larger, thus this species might actually be a closely related *B. cereus*-like species. Furthermore, it the MALDI-TOF factor of 1.99 is low-to-medium as shown, this isolate may represent a variant of a ‘human-friendly’ species *B. atrophaeus*, containing some more common proteins with *B. anthracis*. Within such a closely related ‘*Bacillus* group’, horizontal gene transfer undoubtedly is a common phenomenon. The above analysis points to the possibility of exploring *Bacillus* preparations used for other than human use purposes for valuable species, which eventually could be used for new formulations as human probiotics upon further extensive testing.”

## Funding Update

A correction has been made to Funding. The updated text appears below.

“The work was supported by TECHMATSTRATEG2/410747/11/NCBR/2019 grant to University of Gdansk, Faculty of Chemistry, Molecular Biotechnology Department.”

## Figure/Table Legend

In the original publication, there was a mistake in the legend for *Figure 1*, *Figure 4*, *Figure 5*, *Table 1*. The correct legends appear below.

**Figure 1.** Identification of probiotic bacteria colonies detected in the course of commercial preparation analysis in the growth temperature of 45 °C. Dilution of 10^−7^. Cultures marked: (1) *B. subtilis*, (2) *B. atrophaeus*, (3) *B. cereus*, (4) *B. licheniformis*, (5) *B. pumilus*, (6) *B. amyloliquefaciens*.

**Figure 4.** Inhibition of foodborne pathogenic bacterial growth in the presence of CFS from *Bacillus* strains isolated from potential probiotic source. *Escherichia coli* O157:H7 ATCC 35150 (**A**), *Salmonella Enteritidis* KCCM 12021 (**B**), or *Staphylococcus aureus* KCCM 11335 (**C**) were incubated in the presence of CFS from the *Bacillus* strains (*B. subtilis*, *B. atrophaeus*, *B. cereus*, *B. licheniformis*, *B. pumilus*, *B. amyloliquefaciens*) at 37 °C for 24 h. The bacterial growth was determined at OD_600_. The growth rate of foodborne pathogenic bacteria without CFS was assigned to 100% (Control).

**Figure 5.** Antibiotic resistance and susceptibility of the *Bacillus* derived from a potential probiotic source. Data for triplicate.

**Table 1.** Antibiotic resistance of the *Bacillus* species derived from a potential probiotic source. 

The authors state that the scientific conclusions are unaffected. This correction was approved by the Academic Editor. The original publication has also been updated.
