# Supplementary material for: Analysis of Industrial Bacillus Species as Potential Probiotics for Dietary Supplements
Source: Microorganisms. 2023 Feb 16;11(2):488. doi: 10.3390/microorganisms11020488 (PMC9962517; doi:10.3390/microorganisms11020488)
Supplement: Supplementary file 1 [file microorganisms-11-00488-s001.zip › Supplementary Figure S2.pdf]

Supplementary Figure S2

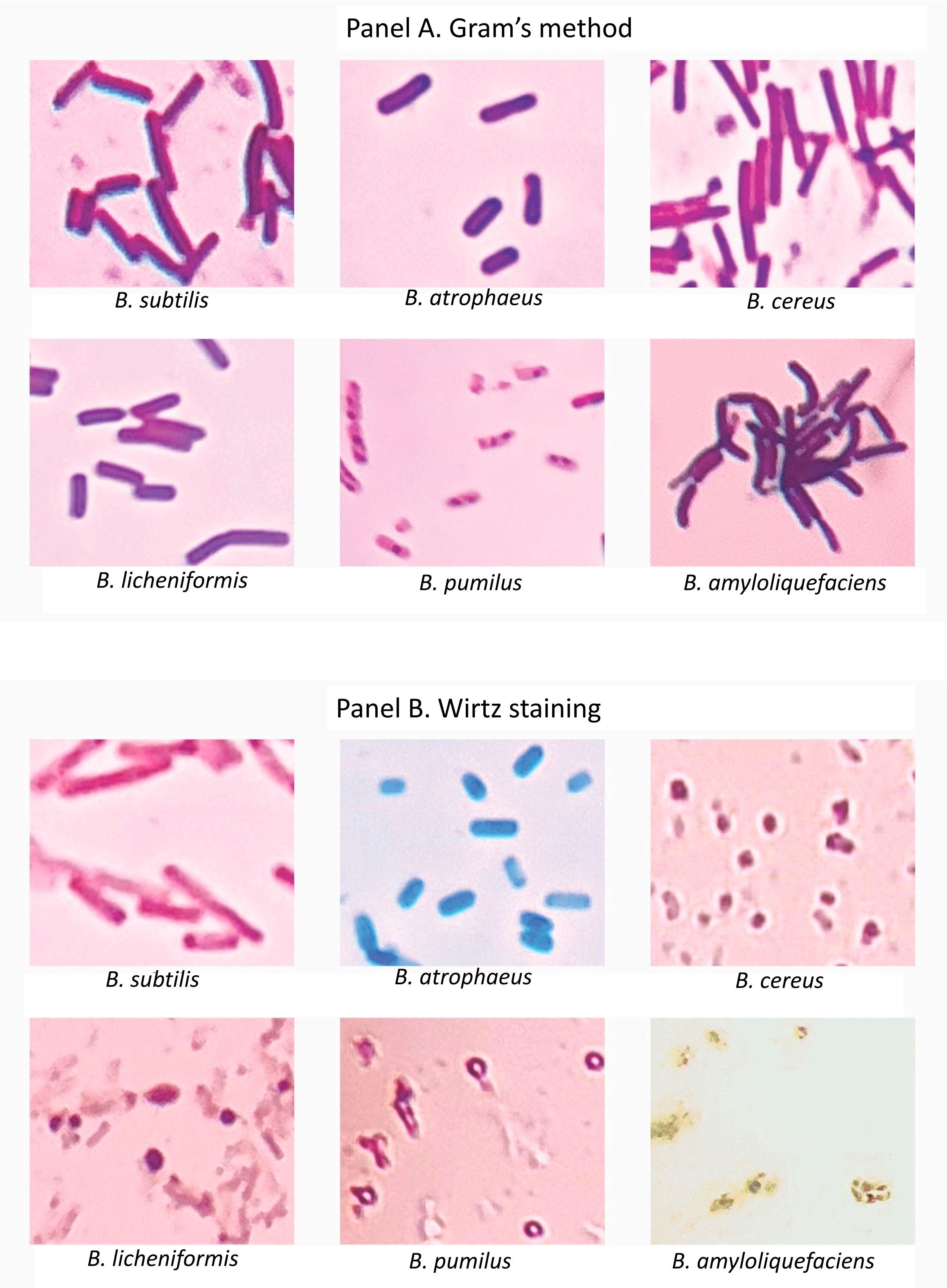

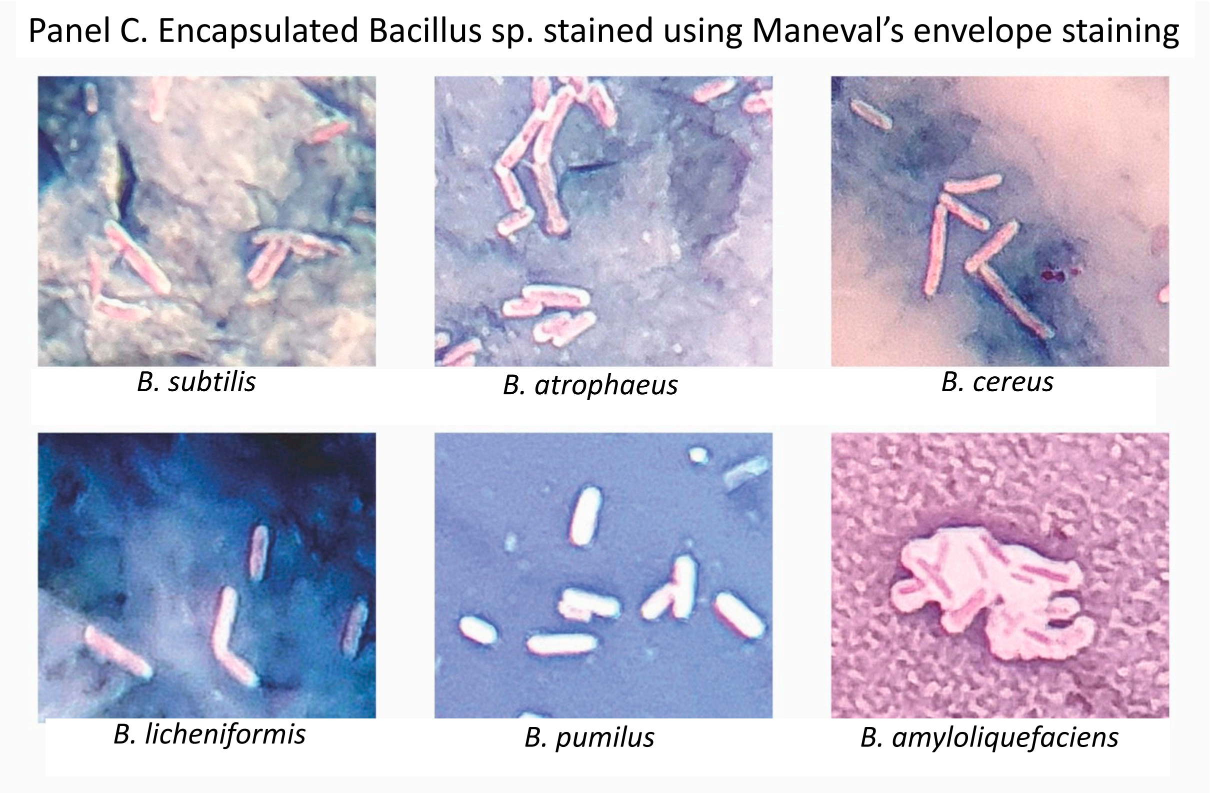

Figure S2. Microscopic analyses of *Bacillus* species cells by Gram staining (panel A), Wirtz staining (panel B) and Maneval tests (panel C)
