# Supplementary material for: Analysis of Industrial Bacillus Species as Potential Probiotics for Dietary Supplements
Source: Microorganisms. 2023 Feb 16;11(2):488. doi: 10.3390/microorganisms11020488 (PMC9962517; doi:10.3390/microorganisms11020488)
Supplement: Supplementary file 1 [file microorganisms-11-00488-s001.zip › Supplementary Figure S3.pdf]

**Supplementary Figure S3**

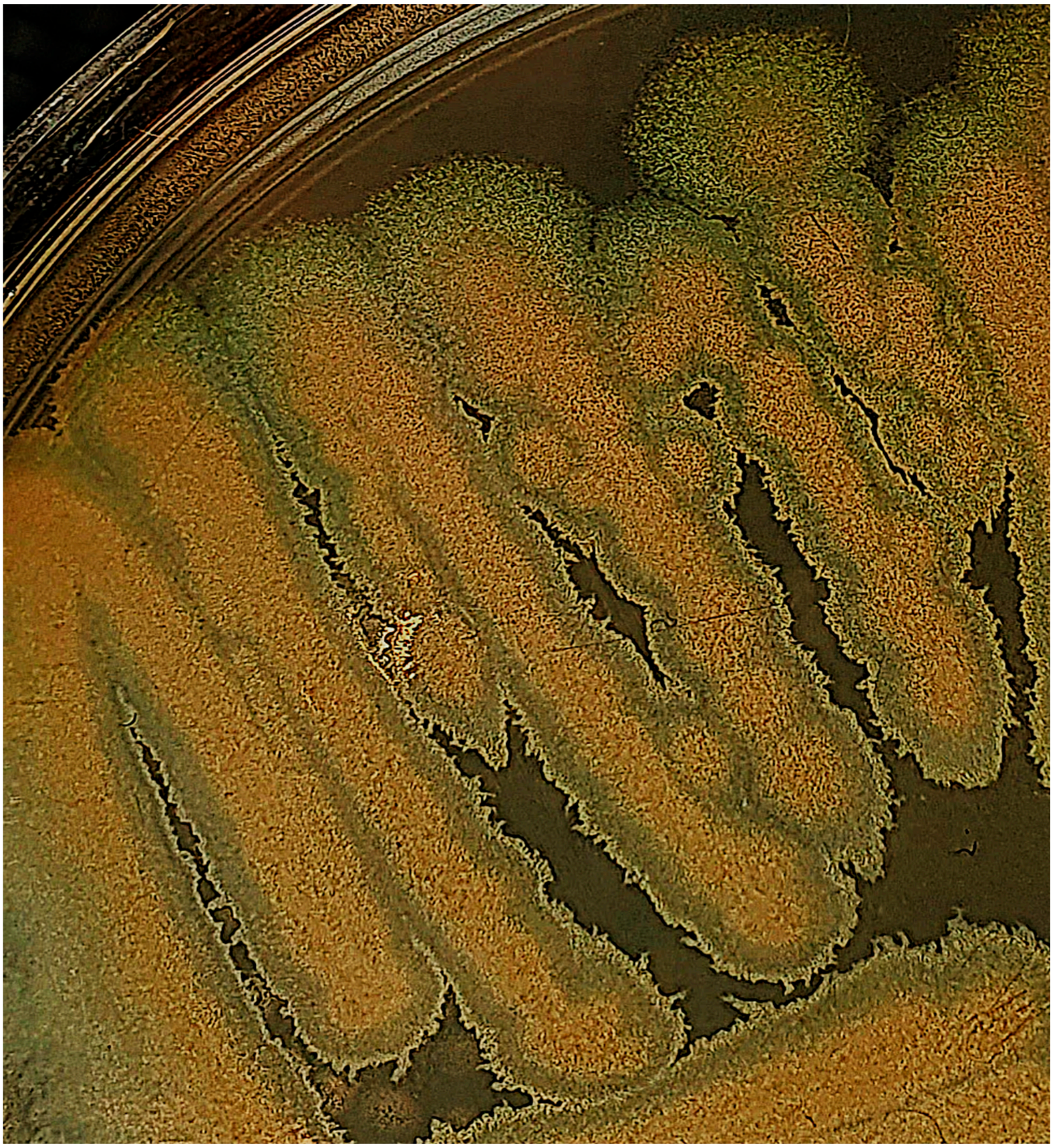

**Figure S3.** The colony of *B. cereus* with a wide zone of hemolysis after incubated in temperature 45°C.
