# Supplementary material for: Analysis of Industrial Bacillus Species as Potential Probiotics for Dietary Supplements
Source: Microorganisms. 2023 Feb 16;11(2):488. doi: 10.3390/microorganisms11020488 (PMC9962517; doi:10.3390/microorganisms11020488)
Supplement: Supplementary file 1 [file microorganisms-11-00488-s001.zip › Supplementary Figure S4.pdf]

**Supplementary Figure S4**

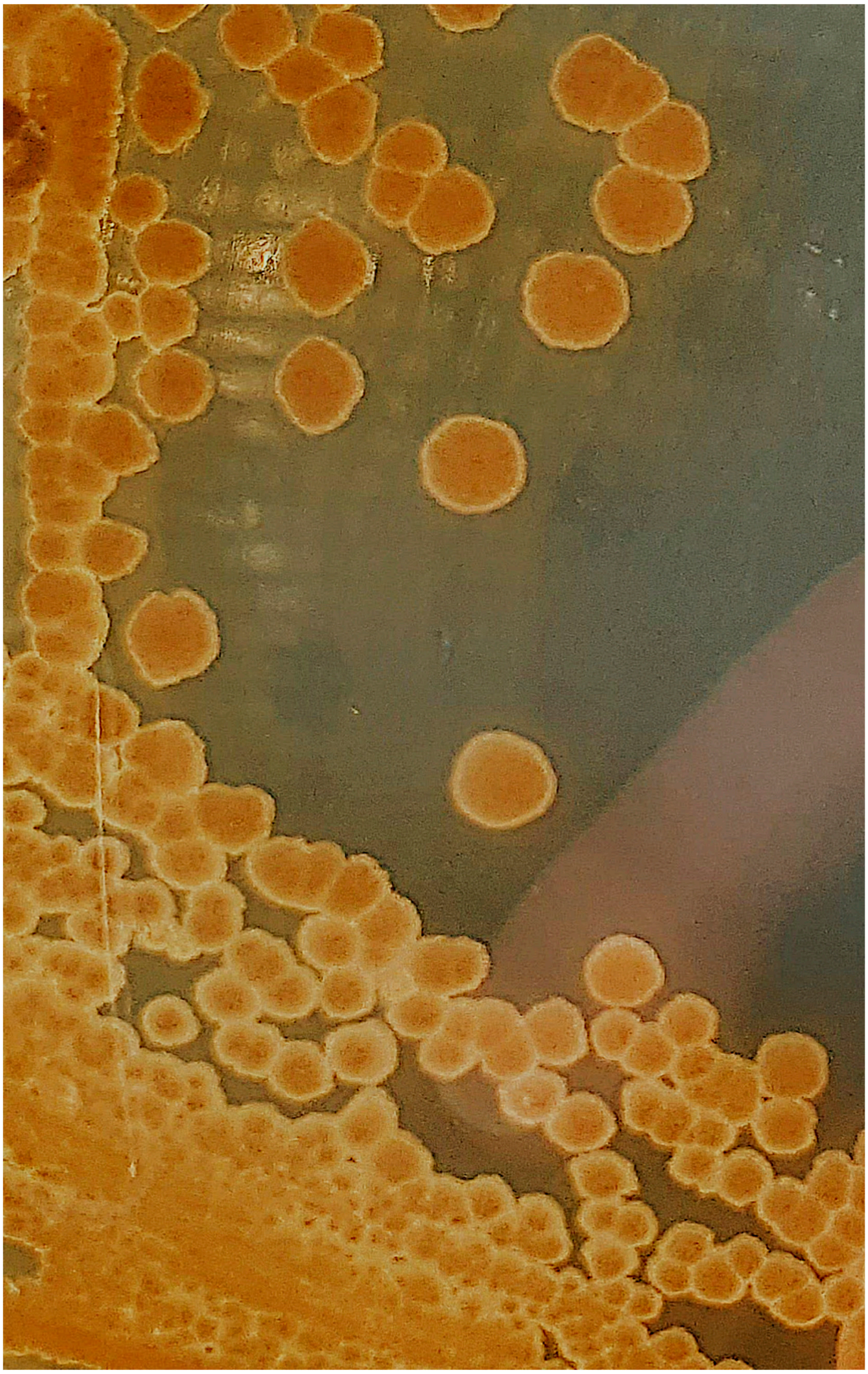

**Figure S4.** Colonies of *B. amyloliquefaciens* with a yellow biofilm secreted into the agar medium.
