# Supplementary material for: Analysis of Industrial Bacillus Species as Potential Probiotics for Dietary Supplements
Source: Microorganisms. 2023 Feb 16;11(2):488. doi: 10.3390/microorganisms11020488 (PMC9962517; doi:10.3390/microorganisms11020488)
Supplement: Supplementary file 1 [file microorganisms-11-00488-s001.zip › Supplementary Figures S5 A-E.pdf]

Supplementary Figures S5 A-E

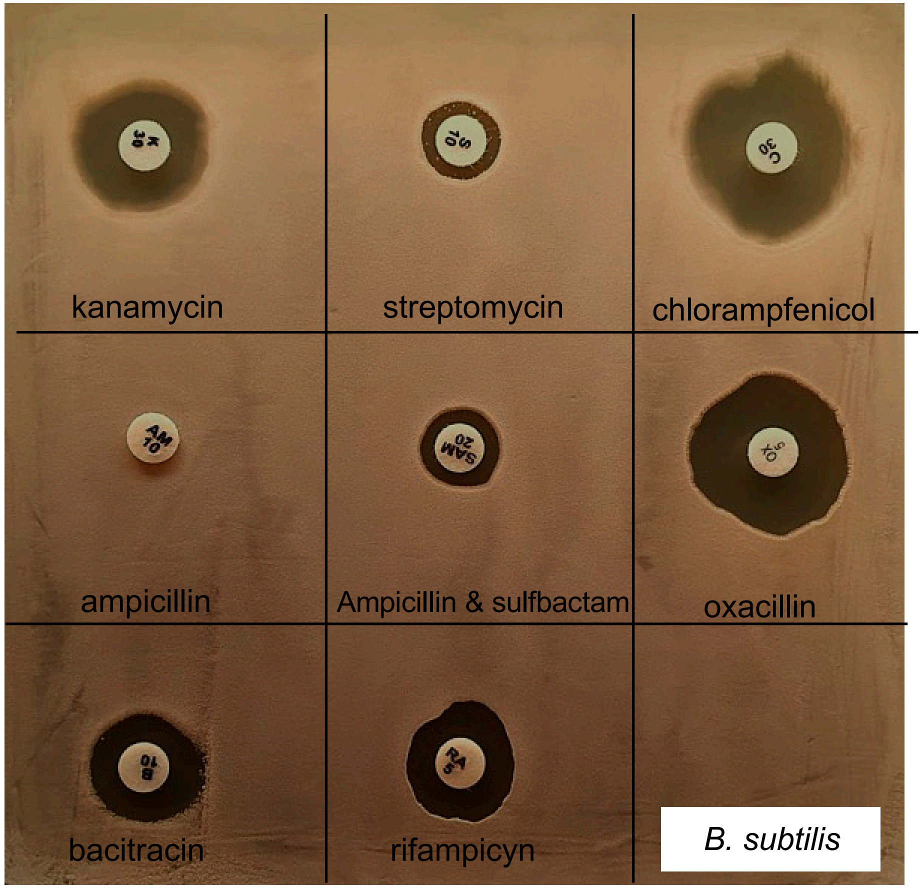

A. Resistance or susceptibility of *B. subtilis* to eight (8) commercial antibiotics discs. Inhibition zone: sensitive, no zone formation: resistant.

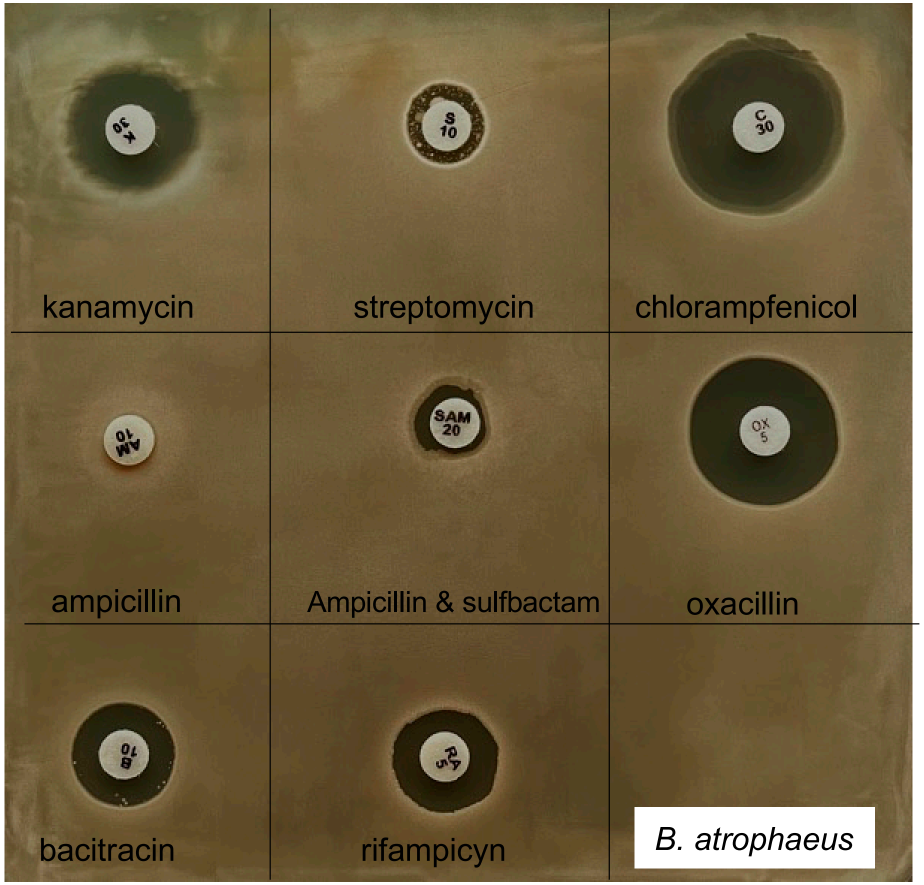

B. Resistance or susceptibility of *B. atrophaeus* to eight (8) commercial antibiotics discs. Inhibition zone: sensitive, no zone formation: resistant.

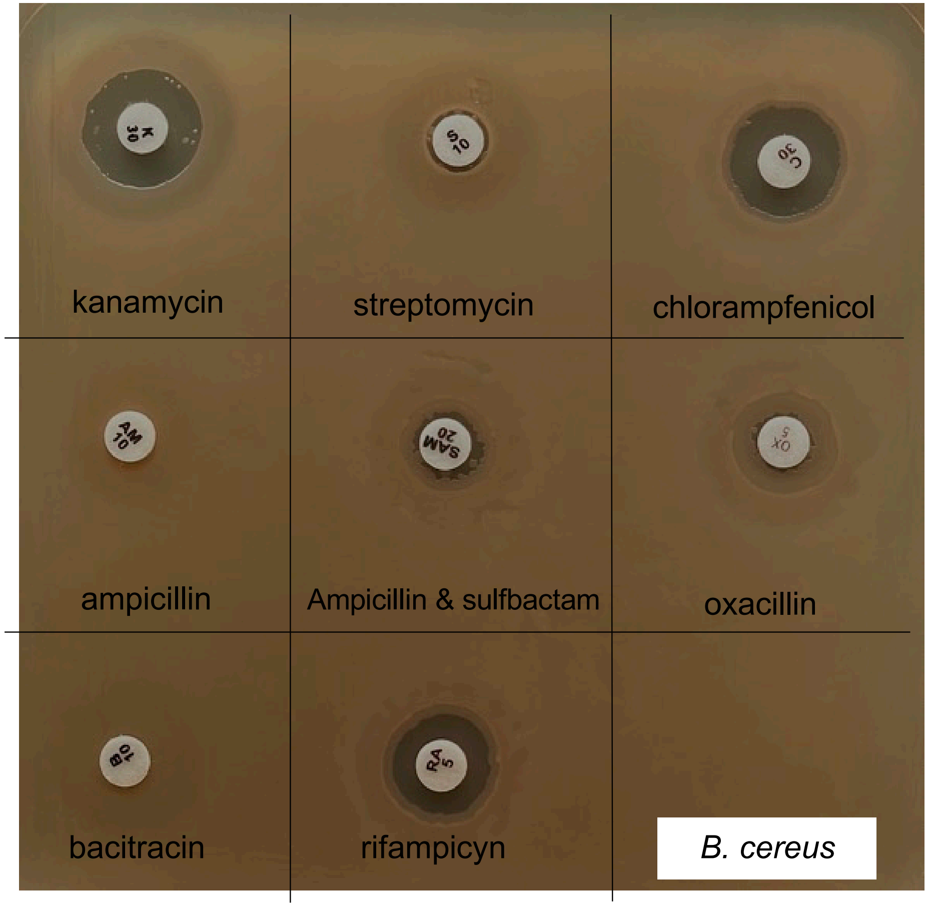

C. Resistance or susceptibility of *B. cereus* to eight (8) commercial antibiotics discs. Inhibition zone: sensitive, no zone formation: resistant.

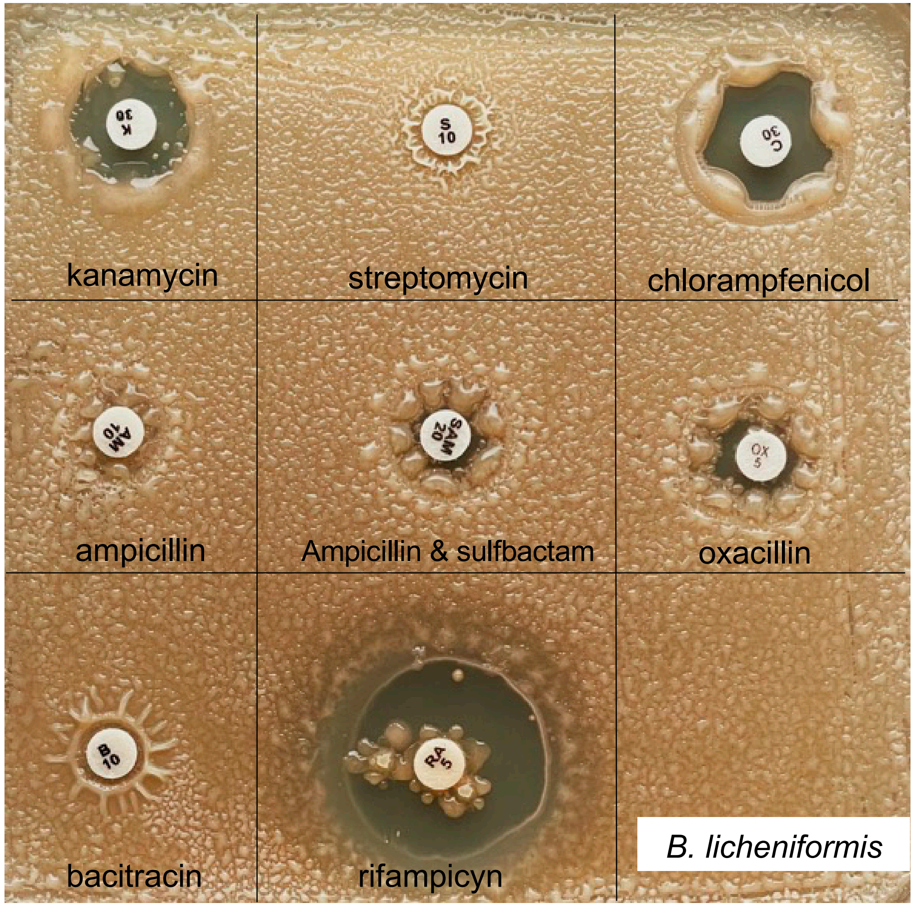

D. Resistance or susceptibility of *B. licheniformis* to eight (8) commercial antibiotics discs. Inhibition zone: sensitive, no zone formation: resistant.

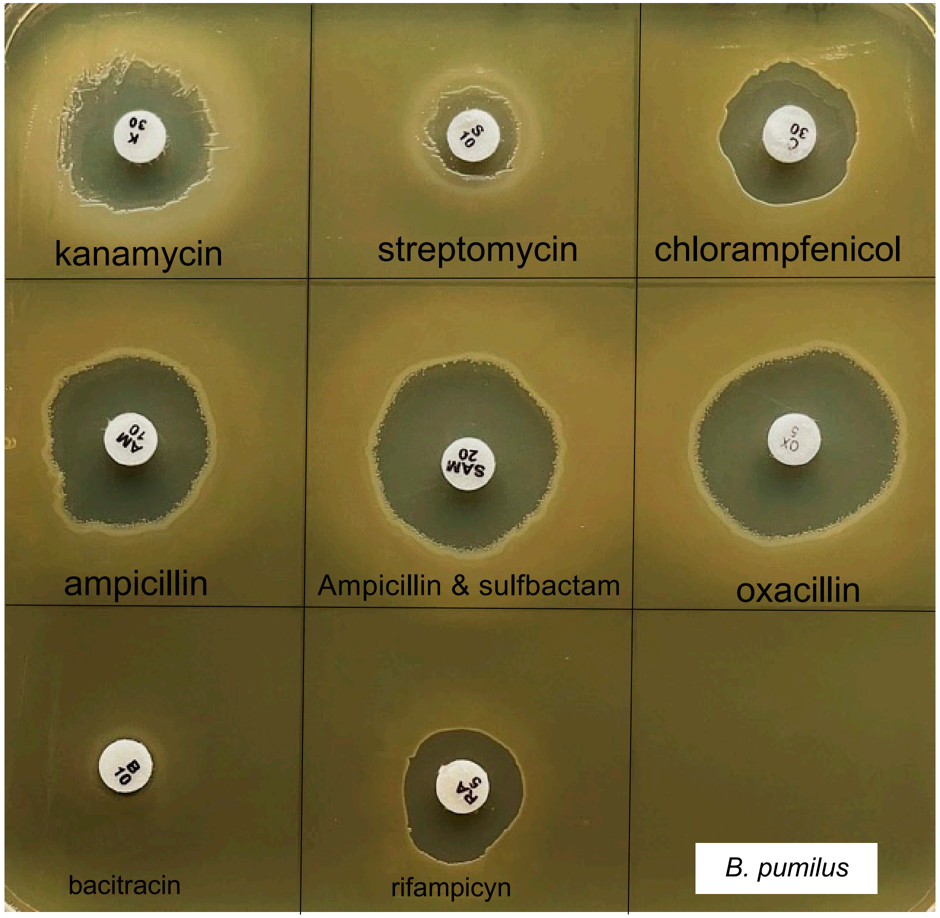

E. Resistance or susceptibility of *B. pumilus* to eight (8) commercial antibiotics discs. Inhibition zone: sensitive, no zone formation: resistant.

Figure S5. Antibiotic sensitivity test of isolated probiotic *Bacillus* derived from the probiotic preparation.
