# Supplementary material for: Analysis of Industrial Bacillus Species as Potential Probiotics for Dietary Supplements
Source: Microorganisms. 2023 Feb 16;11(2):488. doi: 10.3390/microorganisms11020488 (PMC9962517; doi:10.3390/microorganisms11020488)
Supplement: Supplementary file 1 [file microorganisms-11-00488-s001.zip › Supplementary Table S1.pdf]

Supplementary Table S1

Table S1. Quantification of bacterial species in the temperature range tested

**Temperature**	**37°C**	**40°C**	**45°C**	**48°C**	**51°C**	**54°C**	**57°C**
**Number of bacteria species detected**	3	4	6	3	3	2	0
Bacillus **strains identified MALDI-TOF MS method**	*B. subtilis*<br>*B. cereus*<br>*B. licheniformis*	*B. subtilis*<br>*B. atrophaeus*<br>*B. cereus*<br>*B. licheniformis*	*B. subtilis*<br>*B. atrophaeus*<br>*B. cereus*<br>*B. licheniformis*<br>*B. pumilus*<br>*B. amyloliquefaciens*	*B. subtilis*<br>*B. cereus*<br>*B. licheniformis*	*B. subtilis*<br>*B. cereus*<br>*B. licheniformis*	*B. subtilis*<br>*B. cereus*	No strains
**Total number of bacteria counted on the plate with the dilution 10^{-7}**	147	185	93	130	67	239	0
