# Supplementary material for: Analysis of Industrial Bacillus Species as Potential Probiotics for Dietary Supplements
Source: Microorganisms. 2023 Feb 16;11(2):488. doi: 10.3390/microorganisms11020488 (PMC9962517; doi:10.3390/microorganisms11020488)
Supplement: Supplementary file 1 [file microorganisms-11-00488-s001.zip › Supplementary Table S2.pdf]

**Supplementary Table S2**

**Table S2.** The antagonistic activity of CFS from the *Bacillus* strains against foodborne pathogenic bacteria

| ***Bacillus sp.*** | % Inhibition of foodborne pathogenic after 24h | | |
|---|---|---|---|
| | *Escherichia coli* | *Salmonella Enteritidis* | *Staphylococcus aureus* |
| *B. subtilis* | 46.9 ± 0.04 | 56.0 ± 0.01 | 52.3 ± 0.02 |
| *B. atrophaeus* | 54.8 ± 0.03 | 59.0 ± 0.02 | 58.2 ± 0.02 |
| *B. cereus* | 31.0 ± 0.02 | 32.1 ± 0.01 | 30.7 ± 0.01 |
| *B. licheniformis* | 18.9 ± 0.03 | 35.3 ± 0.03 | 24.5 ± 0.02 |
| *B. pumilus* | 50.9 ± 0.03 | 50.9 ± 0.02 | 46.9 ± 0.01 |
| *B. amyloliquefaciens* | 45.9 ± 0.02 | 54.9 ± 0.01 | 47.1 ± 0.04 |
