# Supplementary material for: Analysis of Industrial Bacillus Species as Potential Probiotics for Dietary Supplements
Source: Microorganisms. 2023 Feb 16;11(2):488. doi: 10.3390/microorganisms11020488 (PMC9962517; doi:10.3390/microorganisms11020488)
Supplement: Supplementary file 1 [file microorganisms-11-00488-s001.zip › Supplementary Table S3.pdf]

Supplementary Table S3

Table S3. Zone of inhibition growth of the *Bacillus sp.* derived from probiotic

Bacillus sp.	**Antibiotics**							
	**K**	**S**	**C**	**AM**	**SAM**	**OX**	**B**	**RA**
B. subtilis	14.8 ± 0.3	10.7 ± 5.0	20.2 ± 0.3	0.0 ± 0.0	10.7 ± 0.6	20.2 ± 0.3	12.7 ± 0.6	12.8 ± 0.3
B. atrophaeus	13.7 ± 0.6	8.8 ± 4.0	22.8 ±0.3	0.0 ± 0.0	4.5 ± 2.3	17.3 ±0.3	10.7 ±1.9	13.0 ±0.9
B. cereus	15.5 ± 0.9	7.3 ± 0.6	13.8 ± 0.3	0.0 ± 0.0	8.0 ± 2.0	4.0 ± 1.7	0.0 ± 0.0	12.8 ± 0.3
B. licheniformis	14.0 ± 1.5	0.0 ± 0.0	14.2 ± 0.3	0.0 ± 0.0	6.7 ± 2.3	8.3 ± 1.5	0.0 ± 0.0	23.3 ± 1.5
B. pumilus	17.2 ± 1.0	9.5 ± 0.5	16.8 ± 0.3	20.1 ± 0.1	21.7 ± 0.6	22.8 ± 0.3	0.0 ± 0.0	13.8 ± 0.3
B. amyloliquefaciens	-	-	-	-	-	-	-	-

Results are shown as inhibition diameter of growth of *Bacillus* (in mm) for triplicate samples, with the standard deviation and at a significance level of P< 0.05. Antibiotics discs were as follows: K- kanamycin (30 μg), AM- ampicillin (10 μg), SAM- ampicillin & sulfbactam (20 μg), S- streptomycin (10 μg), RA- rifampicin (5 μg), OX-oxacillin (5 μg), B- bacitracin (10 μg), and C- chloramphenicol (30 μg).
