# Supplementary figures and images for: Analysis of Industrial Bacillus Species as Potential Probiotics for Dietary Supplements
Source: Microorganisms. 2023 Feb 16;11(2):488. doi: 10.3390/microorganisms11020488 (PMC9962517; doi:10.3390/microorganisms11020488)

**Supplementary Figure S1**

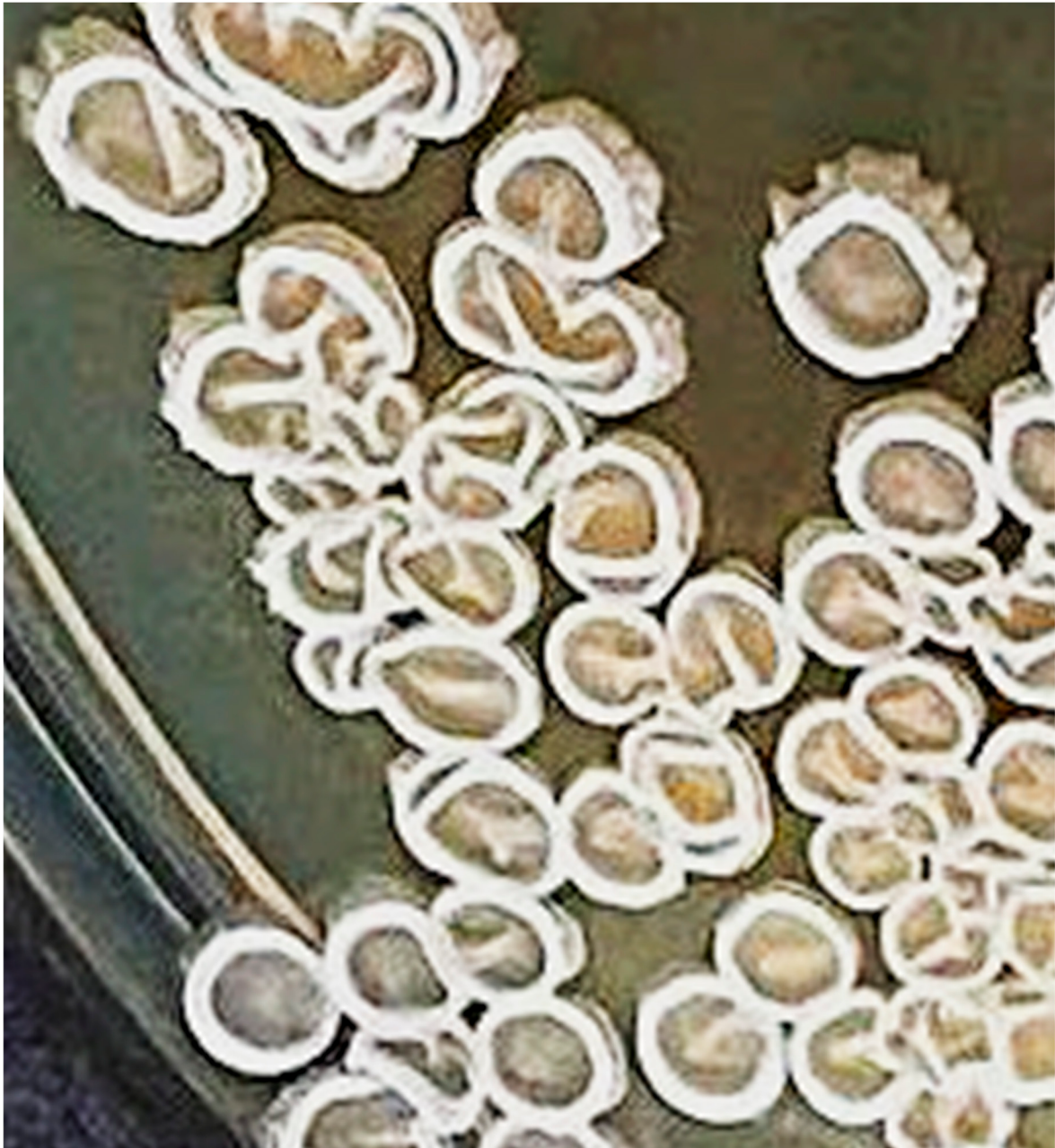

**Figure S1.** Growth of *B. subtilis* on agar plate in 54°C

Supplement: Supplementary file 1 [file microorganisms-11-00488-s001.zip › Supplementary Figure S1.pdf]
